# Citrate Supplementation Modulates Medium Viscosity and Poly‐γ‐Glutamic Acid Synthesis by Engineered *B. subtilis* 168

**DOI:** 10.1002/elsc.70009

**Published:** 2025-03-04

**Authors:** Frederik Völker, Kyra Hoffmann, Birthe Halmschlag, Sandra Maaß, Jochen Büchs, Lars M. Blank

**Affiliations:** ^1^ Institute of Applied Microbiology‐iAMB Aachen Biology and Biotechnology‐ABBt RWTH Aachen University Aachen Germany; ^2^ AVT ‐ Biochemical Engineering RWTH Aachen University Aachen Germany; ^3^ Department of Microbial Proteomics University of Greifswald Greifswald Germany

**Keywords:** *Bacillus subtilis*, biopolymer, co‐feed, ViMOS, viscosity

## Abstract

The industrially attractive biopolymer poly‐γ‐glutamic acid (γ‐PGA) is commonly produced by species of the genus *Bacillus* by co‐feeding different carbon‐ and nitrogen‐sources. Recent studies have highlighted the pivotal role of co‐metabolization of a rapidly degradable carbon source such as glycerol together with citrate for γ‐PGA production, independently fueling biomass generation as well as tricarboxylic acid (TCA) cycle precursor supply. With this study, we report that the sole presence of citrate in the production medium greatly influences growth behavior, γ‐PGA production, and the viscosity of microbial cultures during biopolymer synthesis. Independent of the citrate concentration in the medium, only minor amounts of citrate were imported by *B. subtilis* 168 in the presence of glycerol due to carbon catabolite repression. However, a high citrate concentration resulted in a 6‐fold increase in γ‐PGA titer compared to low exogenous citrate levels. Data suggests that citrate was not used as a precursor in γ‐PGA synthesis but rather influenced the fate of imported glutamate. The citrate concentration also affected medium viscosity as depletion resulted in a remarkable spike in culture broth viscosity. Additionally, cellular proteome analysis at different levels of citrate availability revealed significant changes in protein abundance involved in motility and fatty acid degradation.

*Practical Application:* This research provides critical insights into optimizing γ‐PGA production in *Bacillus subtilis*, particularly by using citrate supplementation to control medium viscosity and improve production yields. The study reveals that citrate not only plays a role in controlling viscosity but also influences intracellular glutamate metabolism, a key factor for γ‐PGA synthesis. Citrate interacts with divalent cations such as Mg^2+^ and Ca^2+^, reducing electrostatic interactions and thus decreasing viscosity in the medium. Additionally, while citrate uptake is limited due to carbon catabolite repression (CCR), even the minimal presence of citrate impacts growth and production. The findings suggest that citrate may trigger unexplored regulatory mechanisms affecting glutamate utilization. Their understanding opens new avenues for industrial optimization, which focus on enhancing glutamate synthesis pathways and exploring novel citrate‐sensing mechanisms. Overall, this research lays the groundwork for improving the efficiency and consistency of γ‐PGA production by fine‐tuning media components and understanding their metabolic effects.

**Abbreviations**
*B. subtilis*
: *Bacillus subtilis*
CcpA: carbon catabolite control protein A
CCR: carbon catabolite repression
Cit: citrate
EDTA: ethylenediaminetetraacetic acid
LFQ: label‐free quantification
OTR: oxygen transfer rate
RAMOS: Respiratory Monitoring Online System
TCA: tricarboxylic acid
ViMOS: Viscosity Monitoring Online System
γ‐PGA: poly‐γ‐glutamic acid

## 1 Introduction

Poly‐γ‐glutamic (γ‐PGA) acid is a homopolymer composed of a variable distribution of D‐ and L‐glutamic acid, linked by a γ‐peptide bond between the α‐amino and γ‐carboxyl groups of the monomers. It is commonly a product of microbial origin [1]. The anionic biopolymer is water‐soluble, biodegradable, biocompatible, and edible and therefore of great industrial interest with suggested applications in food processing, agriculture, medical treatment, hygiene industry, and bioremediation [2, 3, 4]. Each industrial application targets distinct product characteristics, which are largely dependent on the polymers' molecular weight, polydispersity, and enantiomeric composition. Employing microbes for the synthesis of the polymer, these features can be tinkered based on the selected production host, genetic production circuit, and synthesis conditions [5, 6]. Microbial γ‐PGA production has mostly been studied in *Bacillus* and production strains are generally categorized into glutamate‐dependent‐ or independent species, based on their ability to form the biopolymer with or without the supply of exogenous glutamate [7]. Although glutamate‐dependent hosts are reported to produce higher product titers, glutamate supplementation accounts for up to 50% of the production costs [8]. To contribute to a more cost‐efficient bioprocess, glutamate derived from degraded γ‐PGA‐based materials may be used as a substrate, thereby recirculating the biopolymer [9]. Alternatively, metabolic engineering of glutamate‐independent strains together with bioprocess optimizations has been shown to improve the yield of γ‐PGA synthesis [10, 11]. Either strategy calls for a profound understanding of the underlying metabolism and requires further improvements to mature into an industrially feasible implementation. Despite possessing the required set of genes for γ‐PGA production, the common laboratory strain *B. subtilis* 168 is unable to form the biopolymer by nature due to a non‐functional promoter upstream of the γ‐PGA synthesis operon *pgsBCA* [12, 13]. As reported by Halmschlag et al., replacing the native promoter with either the phosphate starvation promoter P_pst_ or the xylose inducible promoter P_xyl_ enables γ‐PGA production, both with or without the presence of exogenous glutamate [14]. However, more detailed investigations on the glutamate (in)dependency of this construct are required to further optimize its productivity.

Recently, γ‐PGA synthesis based on hydrolysates and/or industrial side streams as medium components has been reported [15, 16]. In any co‐feeding approach, the individual *Bacillus* strains used for production are typically confronted with a complex mixture of carbon sources, and their subsequent transport and utilization are governed by carbon catabolite repression (CCR). During CCR, carbon sources transported via the phosphoenolpyruvate‐dependent phosphotransferase system (PTS) such as glucose or sugar alcohols such as glycerol are preferentially metabolized over non‐PTS carbohydrates or organic acids [17]. In particular, the pleiotropic transcription factor CcpA conducts most of the CCR‐mediated response in *B. subtilis*. In the presence of glucose or glycerol, the co‐factor HPr is phosphorylated by the protein kinase HPrK and in response forms a complex with CcpA. This facilitates the binding of the structure to *cre* recognition sites present in promoter regions of various genes and operons, thereby inducing catabolite regulation [18, 19]. In the context of γ‐PGA production, Mitsunaga et al. have first reported the complex interplay between γ‐PGA production and CCR in *B. licheniformis*. They concluded that supplementation of ammonium and citrate to glycerol as the main carbon source is beneficial for the synthesis of the biopolymer, since the addition of nitrogen and citrate individually promotes glutamate production and therefore increases the precursor supply of γ‐PGA [20]. So far, no similar study has been performed in *B. subtilis* 168.

In *B. subtilis* 168, the presence of citrate in the growth medium is sensed by the two‐component system CitST [21]. Similar two‐component systems have been reported for other microorganisms and TCA cycle intermediates and share a common recognition/regulation mechanism [22]. In the case of citrate, the sensor kinase CitS detects the organic acid in the surrounding medium and phosphorylates the effector CitT. CitT then triggers the expression of *citM* encoding for a citrate transporter, by binding to a specific recognition site upstream of the gene. Notably, citrate import and metabolization in *B. subtilis* 168 are prone to regulation by CCR. The promoter region upstream of *citM* contains a *cre* site that is recognized by the HPr‐CcpA complex in the presence of glucose or glycerol. Consequently, the expression of the transporter is blocked, and citrate cannot be internalized [23]. It remains to be explored if a co‐feed of citrate and glycerol promotes γ‐PGA synthesis in *B. subtilis* 168 through changes in metabolism, beyond the reported CCR‐mediated prevention of citrate internalization.

During microbial γ‐PGA production, secretion of the biopolymer into the culture medium gradually increases the viscosity of the broth, which was shown to correlate with the polymer's molecular weight and concentration [24]. Typically, the progression of the medium viscosity is monitored by offline sampling during cultivation, thereby changing the process conditions during the sampling procedure [25]. Moreover, manual sampling offers only a limited time resolution and increased broth viscosity may compromise sampling accuracy. Hoffmann et al. reported online viscosity measurement as a compelling alternative to offline sampling for monitoring γ‐PGA production in a *B. subtilis* 168 derivative [26]. The Viscosity Monitoring Online System (ViMOS) enables simultaneous viscosity tracking of eight shake flasks using non‐invasive optical analysis of the defined rotating liquid position in each flask relative to the direction of the centrifugal acceleration [27]. Additionally, coupling this device to the Respiration Activity MOnitoring System (RAMOS) allows for concurrent online determination of the oxygen transfer rate (OTR) of each culture and therefore results in precise process analysis without offline sampling [28, 29].

This study focused on the effect of citrate supplementation on the γ‐PGA production capabilities of a *B. subtilis* 168 derivative during CCR‐mediated regulation. First, fundamental production parameters such as growth, pH, by‐product synthesis, glutamate consumption, and concentration of the biopolymer were analyzed during cultivation in the presence of glutamate, glycerol, and high/low exogenous citrate concentrations. On‐ and off line analysis tools were used to monitor γ‐PGA synthesis during high‐ and low citrate availability, while also analyzing viscosity development. Moreover, the proteome of cells cultivated in the presence of high/low citrate availability was investigated and used to guide the first approaches of strain engineering. Consequently, deletion strains deficient in citrate sensing/transport were generated and tested concerning the effect of citrate on productivity. With this, the study highlights the distinct effect of citrate supplementation on product titer, molecular weight, and broth viscosity and helps to guide future research approaches towards a more cost‐ and resource‐efficient γ‐PGA production.

## 2 Materials and Methods

### 2.1 Bacterial Strains and Growth Conditions

All strains used in this study are listed in Table 1. All cloning steps were performed in *Escherichia coli* DH5α and the recombinase‐positive strain *E. coli* JM101 was utilized to obtain plasmids suitable for the transformation of *B. subtilis*. As reported earlier, the sporulation‐deficient strain *B. subtilis* Δ*spo*, which is a derivative of the *manPA* negative strain *B. subtilis* IIG‐Bs2 was used as a starting point for genetic manipulation to ensure reproducible growth [5, 30]. To enable γ‐PGA production in this strain background, the xylose inducible promoter P_xyl_ was integrated upstream of the native *pgsBCA* operon. To efficiently use xylose as an inducer, metabolization of the compound was inhibited by the deletion of *xylAB* [38]. For the construction of plasmids and counter selection, strains were cultivated at 37°C in LB medium containing (in litre): 10 g tryptone, 5 g yeast extract, and 10 g NaCl at pH 7.4. Complex media components were bought from Carl Roth GmbH & Co. KG (Karlsruhe, Germany). 100 µg mL^−1^ spectinomycin or 0.5% (w/v) mannose were supplemented when required [30]. For solid plates, 20 g L^−1^ agar was added to the LB medium.

**TABLE 1:** Strains used in this study.

Strain	Genotype/properties	Reference/source
*E. coli* DH5α	*fhuA*2 Δ (*argF‐lacZ*)U169 *phoA glnV*44 Φ80 Δ(*lacZ*)M15 *gyrA*96 *recA*1 *relA*1 *endA*1 thi‐1 hsdR17	[54]
*E. coli* JM101	*glnV*44 thi‐1 Δ(*lac‐proAB*) F'[*lacI^q^ZΔM15 traD*36 *proAB* ^+^]	[55]
*B. subtilis* IIG‐Bs2	ΔSP*β* Δ*skin* ΔPBSX ΔproΦ1 Δ*pks*::*cmR*, ΔproΦ3 *trp*+ Δ*manPA*::*ermR*	[30]
*B. subtilis* ∆*spo*	as IIG‐Bs2 plus *Δbpr ΔsigG ΔsigE ΔspoGA*	[5]
*B. subtilis* ∆*xylAB*	as ∆spo plus Δ*xylAB*	[5]
*B. subtilis* PG10	as ∆*xylAB* plus P_xyl_‐*pgsBCA*	[5]
*B. subtilis* PG10 Δ*citST*	as PG10 plus Δ*citST*	This study
*B. subtilis* PG10 Δ*fecCDEF*	as PG10 plus Δ*fecCDEF*	This study
*B. subtilis* PG10 Δ*citST* Δ*fecCDEF*	as PG10 Δ*citST* plus Δ*fecCDEF*	This study

### 2.2 Construction of the Knockout Mutants

The plasmid required for the deletion of *citST* or *fecCDEF* was assembled using the NEB HiFi DNA Assembly Kit (NEB, Frankfurt am Main, Germany). Two target sequences (TS) with a size of 700 bp flanking the *citST* or *fecCDEF* locus were amplified. For the deletion of *citST*, primers GGGATCACCATCCGTCGCCCggtgcttgtgctgtggat and agcaccagccgtcctttc for TS1 and GAAAGGACGGCTGGTGCTatatgaaaaaatctataatccta and GAGTGATATTATTGACACGCCCccgggaagtcttttatca for TS2 were used (capitalization indicates overhang). To delete *fecCDEF*, primers GGGATCACCATCCGTCGCCCttttacaactggtttcatg and ttatcacatgtaacactataatag for TS1, together with CTATTATAGTGTTACATGTGATAAactcgtcattcttcttatgt and GAGTGATATTATTGACACGCCCcaccctatttttgagaatgt for TS2 were used. Prior to assembly, the vector backbone of the suicide plasmid pJOE‐8739 was linearized using primers gggcgtgtcaataatatcactc and gggcgacggatggtgatccc. The subsequent plasmid construction using the NEB HiFi Assembly Kit was performed according to the manufacturer's protocol. The plasmids were first transformed into chemically competent *E. coli* DH5α cells, confirmed by colony PCR, sequenced, and transformed into *E. coli* JM101. *B. subtilis* PG10 was transformed with the knockout plasmid according to the Paris method [31]. In general, genome editing of *B. subtilis* was performed according to the markerless gene deletion system described by Wenzel & Altenbuchner [30]. In brief, transformants were selected on LB agar plates containing spectinomycin. After this, colonies were first inoculated in LB liquid medium for a period of 8 h and then transferred to LB medium containing 0.5 % (w/v) mannose for counter selection after plasmid loss. After incubation for 16 h, the suspensions were plated on mannose‐containing LB agar and checked for successful plasmid loss by screening for spectinomycin sensitivity. Finally, the deletion of *citST* and/or *fecCDEF* was confirmed by colony PCR.

### 2.3 Shake Flask Cultivation

For γ‐PGA production, the respective strains of *B. subtilis* were first streaked from a cryogenic culture stored at –80°C on LB agar plates and cultivated for 16 h at 37°C. Single colonies were used to inoculate 50 mL of LB liquid medium in 500 mL shake flasks, which were then incubated for 16 h at 37°C while shaking at 200 rpm (25 mm shaking diameter, Infors Multitron, Bottmingen, Switzerland). From this, the main culture was inoculated to a starting optical density at 600 nm (OD_600_) of 0.1, as determined using a Ultrospec 10‐cell density meter (Amersham Bioscience, Little Chalfont, UK). For main cultures, a modified version of the commonly used Medium E was used [20, 32]. This medium contained (in litre): 80 g glycerol, 4 g NH_4_Cl, 0.5 g K_2_HPO_4_, 0.5 g MgSO_4_·7H_2_O, 0.04 g FeCl_3_·6H_2_O, 0.15 g CaCl_2_·2H_2_O, and 0.104 g MnSO_4_·H_2_O. 30 g L^−1^ xylose was added at the beginning of the cultivation to induce expression of *pgsBCA* based on the xylose‐inducible promoter P_xyl_. Depending on the respective experimental run, citrate, and/or L‐glutamate were supplemented accordingly. The pH of the medium was adjusted to 7.4 using 10 M NaOH. Main cultures were then shaken at 300 rpm (50 mm shaking diameter) and 37°C. Experiments on the effect of citrate on growth behavior, γ‐PGA production, OTR, and online/offline viscosity were performed at identical conditions in 250 mL unbaffled shake flasks with a filling volume of 30 mL in quadruplicates. Except for the viscosity analysis of γ‐PGA/citrate/metal‐ion mixtures, all other experiments were performed in 500 mL unbaffled shake flasks with a filling volume of 50 mL, at 300 rpm (25 mm shaking diameter) and in duplicates. The data represents the mean values.

### 2.4 Proteome Analysis

Three biological replicates of *B. subtilis* PG10 were cultivated in the presence of either 1 or 15 g L^−1^ citrate as described in Section 2.3. After 48 h, cells were harvested from 2 mL of culture broth by centrifugation. Afterward, cells were washed three times with ice‐cold PBS (pH 7.5) and stored at –20°C until further use. For analysis, cell pellets were resuspended in 300 µL TE‐Buffer (20 mM Tris‐HCl, 10 mM EDTA, pH 7.5) and added to glass beads (0.1 mm, BioSpec Products, Bartlesville, OK, USA) for disruption using Ribolyser (FastPrep‐24 5G, MP biomedical, Santa Ana, CA, USA) for 30 s and three cycles at 6.5 m/s speed. Afterward, samples were centrifuged at 4°C at 5000 × *g* for 2 min. The resulting supernatant was collected and centrifuged again at 4°C at 10,000 × *g* for 20 min. An aliquot of the obtained supernatants was used for protein determination using a Bradford assay (Roti‐Nanoquant, Roth, Karlsruhe, Germany) according to the manufacturer's instructions. 50 µg of protein was digested using S‐Trap (suspension trap, ProtiFi, Fairport, NY, USA) according to the manufacturer's procedure with slight changes. 2× SDS lysis buffer (10% [w/v] SDS in 100 mM triethylammonium bicarbonate [TEAB], pH 7.5) was added to the samples in a 1:1 ratio. Later, proteins were reduced with 500 mM 1,4‐dithiothreitol (DTT) at 95°C for 10 min and alkylated with 500 mM iodoacetamide (IAA) for 30 min in the dark at room temperature. Samples were acidified with 1.2% (v/v) phosphoric acid, diluted to a ratio of 1:7 with S‐Trap solution (90% [w/v] methanol in 100 mM TEAB), and vortexed thoroughly. Samples were loaded on the S‐Trap microcolumn and washed with S‐Trap solution. Afterward, proteins were digested with trypsin (1:50 ratio) at 47°C for 3 h in a Thermomixer (Eppendorf, Hamburg, Germany). Peptides were eluted from the column using three solvents as follows: 50 mM TEAB, 0.1% (v/v) acetic acid, and 60% (v/v) acetonitrile in 0.1% (v/v) acetic acid.

Peptide mixtures were separated on an Easy nLC 1200 coupled online to an Orbitrap Elite mass spectrometer (ThermoFisher Scientific, Waltham, MA, USA). In‐house self‐packed columns (i.d. 100 µm, o.d. 360 µm, length 200 mm) packed with 3.0 µm Dr. Maisch Reprosil C18 reversed‐phase material (ReproSil‐Pur 120 C18‐AQ) heated to 45°C were loaded with 0.1% (v/v) acetic acid at a maximum pressure of 500 bar. Peptide elution was performed in a 180 min non‐linear gradient from 1% to 99 % solvent (0.1% [v/v] acetic acid in 95% [v/v] acetonitrile) at a constant flow rate of 300 nL/min. Eluted peptides were measured in the Orbitrap with a resolution of R = 60,000 with lock‐mass correction activated. Following each MS‐full scan, up to 20 dependent scans were performed in the linear ion trap after collision‐induced dissociation fragmentation (CID) based on the precursor intensity.

Database search against a database of *B. subtilis* 168 downloaded from Uniprot (date 15/07/2024, UP000001570, 4264 entries), as well as label‐free quantification (LFQ), was performed using MaxQuant (version 2.5.0.0) [33]. Common laboratory contaminants and reversed sequences were included by MaxQuant. Search parameters were set as follows: peptide tolerance, 4.5 ppm; min fragment ions match per peptide, 2; primary digest reagent, trypsin; missed cleavages, 2; variable modifications, oxidation M (+15.9949), acetylation N, K (+42.0106); fixed modification, carbamidomethyl C (+57.0215). Match between runs was enabled with default parameters. Results were filtered for a 1% false discovery rate (FDR) on the spectrum, peptide, and protein levels. A minimum of two unique peptides were required for protein identification. Normalized LFQ was used for relative quantification of the identified proteins with a minimum of two valid values per condition [34]. Fold changes were calculated from averaged log2‐transformed LFQ intensities by subtracting the average value of condition 1 (high citrate availability) from the average value of condition 2 (low citrate concentration). Significance was considered for log_2_fold change>|0.8| and *p* value < 0.01.

The mass spectrometry proteomics data have been deposited to the ProteomeXchange Consortium via the PRIDE partner repository with the dataset identifier PXD058048 [35].

### 2.5 Determination of OTR, Online‐ and Offline‐Viscosity

Online analysis of the OTR and viscosity of main cultures at high (15 g L^−1^) or low (1 g L^−1^) exogenous citrate levels using the ViMOS device coupled to a RAMOS system was realized as described previously [14, 26–29]. Commercial versions of the RAMOS device are available at either Kuhner AG, Birsfelden, Switzerland, or HiTech Zang, Herzogenrath, Germany.

For validation of the online viscosity data attained during γ‐PGA production, the viscosity of offline samples was analyzed as well. The viscosity was determined using a Physica MCR 301 rheometer (Anton Paar Germany GmbH, Ostfildern‐Scharnhausen, Germany) in a range of shear rates between 100 and 5000 s. The device was operated with a cone‐plate measuring system, also from Anton Paar (cone CP50‐0.5/TG with a cone diameter of 49.945 mm, a cone angle of 0.467 and a cone truncation of 54 µm; plate P‐PTD200/TG+H‐PTD200). For the evaluation of the attained datasets, the software RheoPlus/32 V3.40 (Anton Paar Germany GmbH, Ostfildern‐Scharnhausen, Germany) was used. The offline viscosity was analyzed at 37°C with a sample volume of approximately 480 µL. As the biopolymer exhibits pseudoplastic properties, the effective viscosity in shake flasks depends on the specific shear rate observed at the specific sampling point. Consequently, the effective viscosity and shear rates were calculated as described by Giese et al. [36].

### 2.6 γ‐PGA Quantification With CTAB

Biopolymer quantification was performed using a turbidity assay based on the interaction of γ‐PGA with cationic cetyltrimethylammonium bromide (CTAB) [37]. Briefly, samples withdrawn from growing cultures were diluted 1:10 in distilled water and cells were subsequently separated by centrifugation (30 min, 16,000 g). Three times the sample volume of pure ethanol was added to the collected supernatant to precipitate the biopolymer. The precipitate was then resuspended in distilled water and used for further analysis. The γ‐PGA sample was diluted to fit the linear measuring range of the assay between 0.01 and 0.1 g L^−1^ of γ‐PGA and then mixed in equal amounts with 0.07 M CTAB solution dissolved in 2% (w/v) NaOH. The mixture was incubated for 3 min at room temperature, whereupon the turbidity was measured at 400 nm using a Synergy MX microplate reader (BioTek Instruments, Winooski, USA). The results were compared to a calibration curve based on a 1 MDa γ‐PGA standard (Henkel AG & Co. KGaA, Düsseldorf).

### 2.7 γ‐PGA Molecular Weight Determination With GPC

The molecular weight of γ‐PGA produced during the cultivations was analyzed using gel permeation chromatography (GPC) as previously demonstrated [26, 38]. The analytical system (HLC‐8320GPC) was operated with a TSKgel GMPWxl column (300 × 7.8 mm, Tosoh Bioscience GmbH, Stuttgart, Germany) combined with a TSKgel PWxl guard column (40 × 6 mm, Tosoh Bioscience GmbH, Stuttgart, Germany). 30 mM KNO_3_ with a flow rate of 1 mL/min was used as the mobile phase, while the GPC system was maintained at 40°C. Cell‐free γ‐PGA containing supernatant was collected as described for CTAB quantification but was additionally filtered through Rotilabo syringe filters (0.22 µm, CA). 30 µL of the filtrate was subsequently injected into the system for molecular weight determination. Poly(styrene sulfonate) sodium salt standards (PSS Polymer Standards Service GmbH, Mainz, Germany) with a peak molecular weight of 4.21–976 kDa were used to generate a calibration curve for data analysis.

### 2.8 Analysis of Carbon Sources, By‐Products, and Glutamate

The concentration of glycerol, citrate, acetate, acetoin, and 2,3‐butanediol was determined using high‐performance liquid chromatography (HPLC‐UV/RI). The DIONEX UltiMate 3000 HPLC System (Thermo Scientific, Waltham, MA, USA) was equipped with a Metab‐AAC column (300 × 7.8 mm column, ISERA, Düren, Germany). 5 mM H_2_SO_4_ was used as mobile phase with an isocratic flow of 0.6 mL/min at a temperature of 65°C for 23 min. Cell‐free supernatant was filtered through Rotilabo syringe filters (0.22 µm, CA) and 5 µL of the filtrate was injected into the system. The respective metabolites were detected using a DIONEX UltiMate 3000 Variable Wavelength Detector set to 210 nm and a RefractoMax 521 RI detector (Thermo Scientific, Waltham, MA, USA), and their concentration was quantified based on calibration curves established for all standards. Glutamate in cell‐free culture supernatants was analyzed using a 4BioCompact metabolite analyzer (4BioCell, Bielefeld, Germany) equipped with a glutamic acid assay kit according to the manufacturer's protocol.

### 2.9 Viscosity Analysis of γ‐PGA/Citrate/Metal Ion Mixtures

The offline viscosity of a mixture of 5 g L^−1^ γ‐PGA, 0, 1, or 15 g L^−1^ citrate, and Ca^2+^, Mg^2+^, or a combination of both was analyzed as portrayed in 2.5. To supply metal ions to the mixture, CaCl_2_·2H_2_O and MgSO_4_·7H_2_O in equal concentrations as present in the used cultivation medium were supplemented. The mixtures were adjusted to a volume of 1 mL with distilled water and cultivated in System Duetz 24 deep‐well microtiter plates (EnzyScreen, Heemstede, the Netherlands) at 37°C, 200 rpm (25 mm shaking diameter) for 16 h prior to viscosity analysis. The γ‐PGA with a purity of 95% and a molecular weight of 700 kDa (according to the supplied data sheet) used in this experiment was purchased from ChemScene (Cat# CS‐0043360, New Jersey, USA).

## 3 Results

### 3.1 Influence of Citrate on Cultivation‐ and γ‐PGA Production Parameters

**FIGURE 1:**
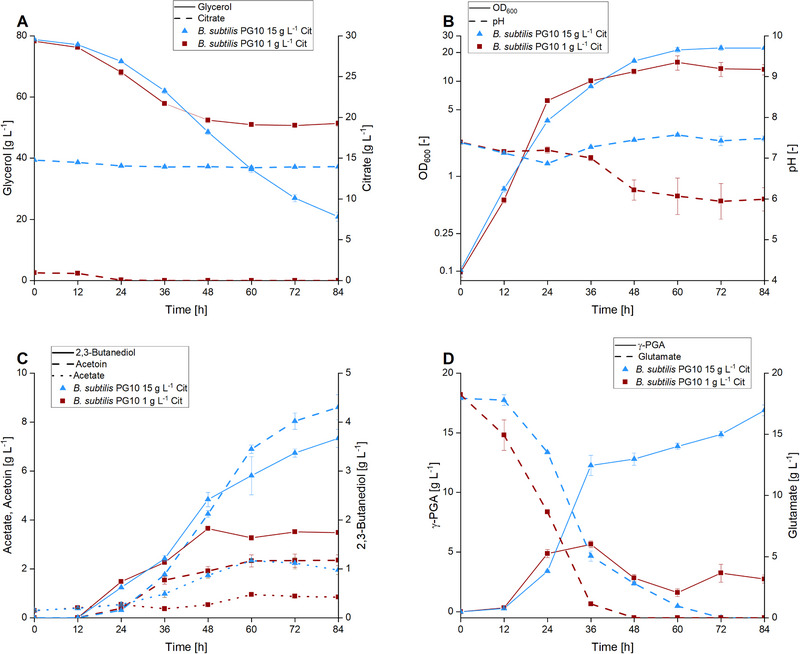
Quantitative physiology of *B. subtilis* PG10 at high and low citrate concentrations. Carbon source depletion (A), growth and pH development (B), synthesis of by‐products (C), progression of γ‐PGA titer and glutamate consumption (D) of the xylose‐inducible promoter harboring strain *B. subtilis* PG10 in the presence of 20 g L^−1^ glutamate and high (15 g L^−1^) or low (1 g L^−1^) levels of exogenous citrate (Cit) is shown. The experiment was performed in 250 mL shake flasks with 30 mL filling volume, at 300 rpm (25 mm shaking diameter) and 37°C. The experiments were performed in quadruplicates, error bars represent the standard deviation.

The domesticated laboratory strain *B*. *subtilis* 168 is frequently applied to study fundamental metabolic processes due to its well‐characterized nature, fully sequenced genome, and ease of genetic transformation. In a previous study, this strain was further optimized; for instance, its ability to sporulate was removed, and a counter‐selection system was established to further simplify genetic modifications [5]. However, this parental *B. subtilis* 168 derivative (*B. subtilis* Δ*spo*) lacks a functional promoter upstream of the γ‐PGA synthesis genes (*pgsBCA*). To address this, the native promoter was replaced with the xylose‐inducible promoter P_xyl_ and the xylose‐utilization operon *xylAB* was removed to prevent inducer degradation. The resulting strain *B. subtilis* PG10 combines the advantage of easy handling with the ability of γ‐PGA synthesis, enabling recently published γ‐PGA titers of up to 10 g L^−1^ while facilitating the study of associated metabolic pathways [14]. To further improve biopolymer production, increased availability of the γ‐PGA precursor glutamate independent of metabolic engineering of the host was targeted by co‐feeding citrate, which was reported to amplify glutamate replenishment in *B. licheniformis* [39]. Therefore, *B. subtilis* PG10 was cultivated in a medium supplemented with either 1 g L^−1^ (low) or 15 g L^−1^ (high) of citrate, and the impact of the organic acid on culture pH, biomass formation, carbon source depletion, and biopolymer production was investigated (Figure 1). Preparation of the cultivation medium without citrate was attempted by adding ethylenediaminetetraacetic acid (EDTA) as an alternative chelator but did not result in an applicable medium due to the precipitation of medium components. During the first 24 h of the cultivation, the citrate concentration decreased by 1 g L^−1^ in all tested setups, resulting in either complete depletion of extracellular citrate or a residual amount of 14 g L^−1^ (Figure 1A). In the case of the latter, no further reduction in concentration was observed after the initial decrease. *B. subtilis* PG10 grown in the presence of high citrate levels continuously consumed the available glycerol up to a total of 58 g L^−1^ within 84 h, whereas only 28 g L^−1^ of the carbon source was used in setups with low citrate supply. Interestingly, in the first 36 h of the experiment, faster consumption of the available glycerol was observed in the presence of low citrate levels as compared to setups with high citrate availability. Following this, a distinct reduction in consumption rate was observed in setups with a low initial citrate concentration with no further glycerol metabolization after 48 h (Figure 1A). This observation is also reflected in the growth profile of the strain, as a higher OD_600_ was reached in the first 36 h of the experiment when facing low citrate concentration. After this point, *B. subtilis* PG10 grown at high citrate availability surpassed the other approach in growth, reaching a maximum OD_600_ of 21 after 60 h. In comparison, a maximum OD_600_ of 15 was reached after 60 h by the strain cultivated at low citrate levels. Apart from an initial decrease in the pH of cultures supplemented with high levels of citrate, a constant pH of 7.4 was observed during the experiment. However, in setups with low citrate levels, the pH decreased from 7.4 to a constant pH of 6 after 48 h of cultivation (Figure 1B). However, it was shown that *B. subtilis* 168 produces γ‐PGA even at a pH below 6 [14]. Therefore, it is unlikely that the production arrest was observed due to a decrease in pH. A common observation during the cultivation of *B. subtilis* 168 under carbon excess is the accumulation of overflow metabolites such as 2,3‐butanediol, acetoin, and acetate. In this experiment, higher concentrations of each by‐product were accumulated in the presence of high citrate levels, with only minor amounts present in setups with low initial citrate concentrations. In the case of the latter, even with elevated glycerol consumption within the first 36 h of cultivation, fewer by‐products were detected (Figure 1C). Surprisingly, citrate supplementation had a significant impact on γ‐PGA titer and glutamate consumption. After 36 h, *B. subtilis* PG10 produced a γ‐PGA titer of 12.3 g L^−1^ which further increased to 16.9 g L^−1^ within the next 48 h when cultivated in the presence of high citrate availability. In this setup, the biopolymer was synthesized with a rate of 0.74 g L^−1^ h^−1^ between 24 and 36 h, the highest rate observed in the experiment. Within the next 48 h, the rate of production decreased significantly to 0.096 g L^−1^ h^−1^. In contrast, γ‐PGA production in medium with low citrate levels reached only a maximum titer of 5.7 g L^−1^ after 36 h of cultivation, even though at this time, more glycerol was being consumed as in setups with high citrate availability. Within the next 48 h, the biopolymer titer subsequently decreased to 2.7 g L^−1^ indicating the onset of γ‐PGA degradation. The highest rate of γ‐PGA production in this setup was observed between 12 and 24 h of cultivation with a rate of 0.4 g L^−1^/h. In the case of high citrate availability, glutamate consumption followed the course of γ‐PGA synthesis, showcasing a major reduction in extracellular glutamate concentration within the first 36 h, coinciding with the point of highest biopolymer production rate (Figure 1D). Notably, strains presented with low exogenous citrate concentration consumed the available glutamate faster than strains grown in the high‐citrate condition. However, no correlation between consumption of available glutamate and biopolymer synthesis was observed in the presence of low citrate levels.

### 3.2 Viscosity Profiling in Presence of Different Citrate Concentrations

**FIGURE 2:**
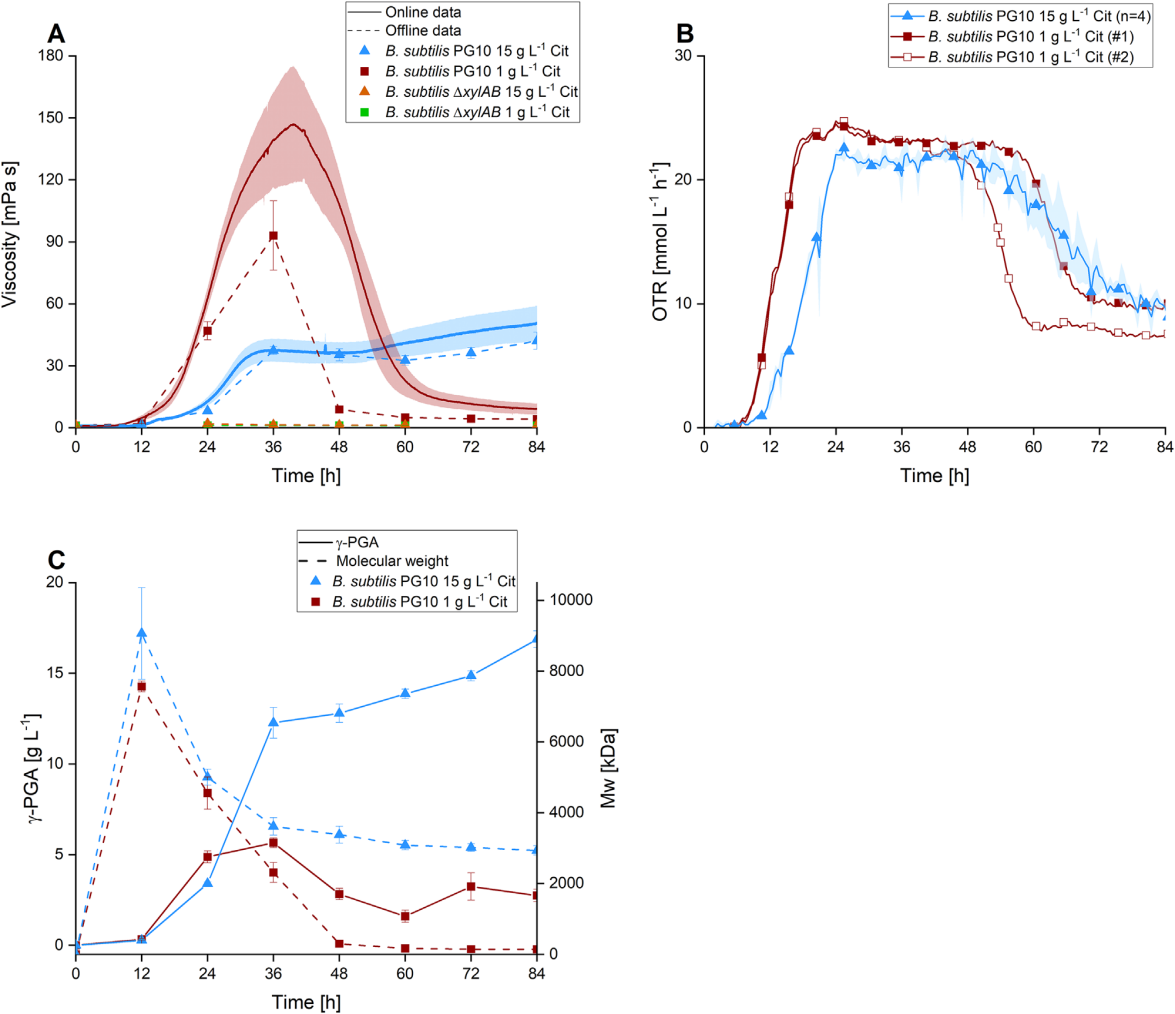
Online profiling of viscosity and OTR of *B. subtilis* PG10 during γ‐PGA production. The development of the broth viscosity of cultures of *B. subtilis* PG10, carrying the xylose‐inducible promoter P_xyl_ cultivated in the presence of 20 g L^−1^ glutamate and either high (15 g L^−1^) or low (1 g L^−1^) levels of exogenous citrate (Cit) was analyzed by either online‐ or offline‐analytics. Cultures of *B. subtilis* Δ*xylAB* carrying the native *pgsBCA* promoter were analyzed as control (A). Simultaneously, the oxygen transfer rate (OTR) of the respective cultures was measured (B). The γ‐PGA titer presented in Figure 1 is shown as a reference, together with the molecular weight of the polymer (C). The experiment was performed in 250 mL shake flasks with 30 mL filling volume, at 300 rpm (25 mm shaking diameter) and 37°C. The experiments were performed in quadruplicates. In the case of online‐ and offline‐viscosity and OTR collected in the presence of high citrate availability, the data represents the mean value of all replicates with error bars indicating the standard deviation. In the presence of low levels of citrate, the shown OTR data represents the results obtained from two individual replicates.

While conducting experiments to examine γ‐PGA synthesis at different levels of extracellular citrate, a notable change in the viscosity of the culture broths was perceived. This observation was further explored by using the ViMOS device for online viscosity analysis, mimicking shaking frequency‐ and diameter, temperature, flask size, and filling volume of the offline experiment presented in Figure 1. The viscosity of samples collected during this cultivation was measured offline as a control to validate the ViMOS online data (Figure 2A). At high citrate concentrations, the viscosity increased mainly between 24 and 36 h, which also resembled the range of the highest γ‐PGA production rate (see Figure 2C), and continued to increase gradually until the end of the experiment. However, at low citrate availability, the viscosity started to increase after 24 h or at citrate depletion and reached a sharp peak in viscosity after 40 h. Here, the viscosity is nearly 4× as high as in the condition with high amounts of exogenous citrate, even though the γ‐PGA titer and molecular weight were significantly lower at this time point (see Figure 2C). The offline viscosity data of cultures with high citrate levels is in good agreement with the online data. In case of low citrate availability, the offline data portrays a lower overall viscosity shortly before and after reaching peak online viscosity. However, both online‐ and offline results depict the same trend of a remarkable viscosity increase during the start of the cultivation, followed by a rapid viscosity decrease. No viscosity increase was observed during the cultivation of the control strain *B. subtilis* Δ*xylAB*. Consequently, there is no obvious correlation between viscosity and γ‐PGA‐dependent parameters in configurations with low citrate levels, indicating a connection between citrate availability and medium viscosity as citrate is the only parameter which is different in both setups.

Simultaneously, the online OTR was captured to monitor the metabolic activity of the individual cultures during γ‐PGA synthesis (Figure 2B). The OTR is often used as a proxy to follow cell growth and metabolic activity, allowing us to compare the offline data presented in Figure 1A with the online data shown in Figure 2B. The experiments were performed in four independent flasks for each tested setup. However, the high viscosity profile observed at low citrate levels resulted in strong fluctuations in the measured OTR signal in two flasks, which is why for this condition only the two flasks with evaluable data are presented. This phenomenon of OTR inequality among flasks during the same condition was already depicted in earlier works [40]. In the presence of low citrate levels, an increase in OTR was detected after 7 h, reaching a maximum OTR of 24.2 mmol/L/h after 24 h. From there on, the OTR reached a plateau and decreased sharply after 48 and 57 h, respectively. Interestingly, the OTR signal did not fully decline but stabilized at 9.8 and 7.5 mmol/L/h until the end of cultivation. In the case of high citrate availability, the OTR signal increased after 10 h to a maximal OTR of 22.6 mmol/L/h, plateaued, and gradually decreased after 52 h to an OTR of 9.7 mmol/L/h at the end of cultivation. For low citrate availability, no further increase in OD_600_ was observed after 60 h (Figure 1B), which agrees with the observed decrease in OTR. In the case of a high citrate concentration, no further growth was observed after 60 h as well, however, the strain continued to utilize the available glycerol until the end of the experiment, which is also depicted by the OTR signal. Here, the signal decreased gradually instead of dropping sharply, as in the case of low citrate concentrations.

**FIGURE 3:**
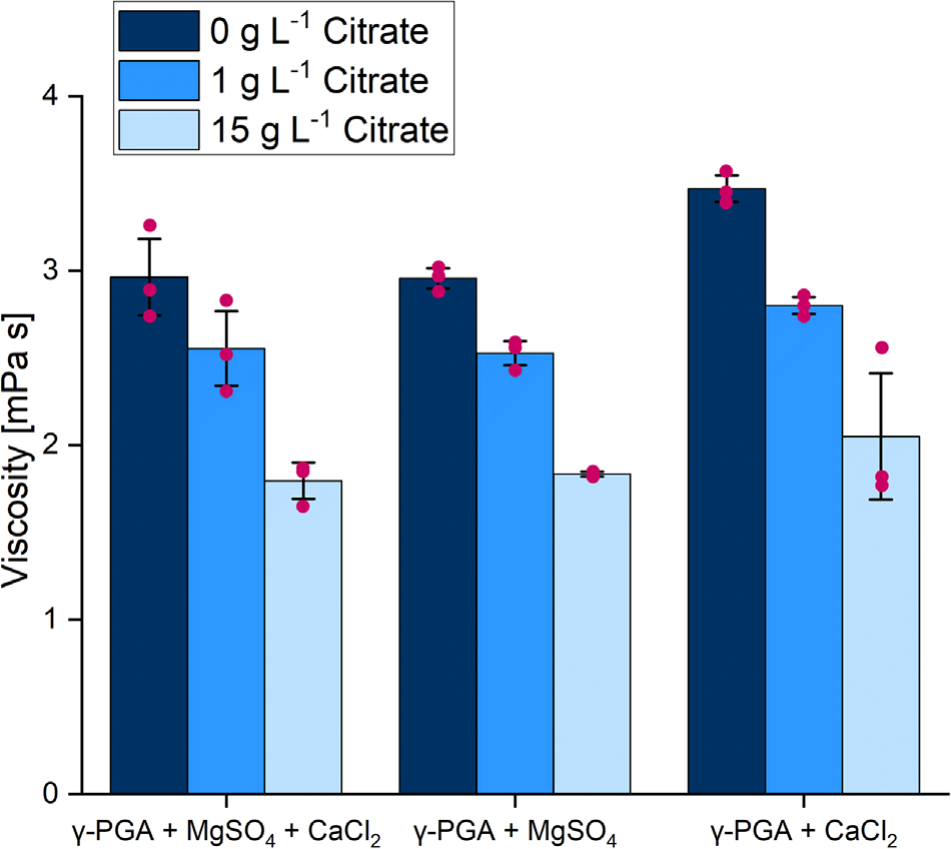
In vitro viscosity of γ‐PGA/metal salt mixtures. The offline viscosity of a γ‐PGA standard was analyzed in combination with CaCl_2_, MgSO_4,_ or a combination of both in the absence of citrate or in the presence of 1 or 15 g L^−1^ of citrate. The experiment was performed in 24 deep‐well System Duetz plates with a filling volume of 1 mL, cultivated at 200 rpm (shaking diameter 25 mm) and 37°C. The experiment was performed in triplicates, error bars represent the standard deviation.

Citric acid is known as a prominent chelator for divalent metal cations and is required as a stabilizing agent for Fe(III) in microbial cultivation media [41]. During γ‐PGA production, the observed increase in broth viscosity in configurations with low citrate levels coincided with the complete removal of citrate from the cultivation medium by internalization into the production host (Figures 1A and 2A). To investigate this hypothesis in more detail, the interaction of γ‐PGA, citrate, and metal ions at equal concentrations, as present in the cultivation medium, was tested in vitro. Without the addition of citrate, mixtures of γ‐PGA with MgSO_4_, CaCl_2_, or a mixture of both showed the highest viscosity. However, the viscosity gradually decreased upon the addition of citrate, indicating the successful withdrawal of cations bound to γ‐PGA by chelation with citrate (Figure 3).

### 3.3 Proteome Changes in *B. subtilis* 168 during γ‐PGA Synthesis

**FIGURE 4:**
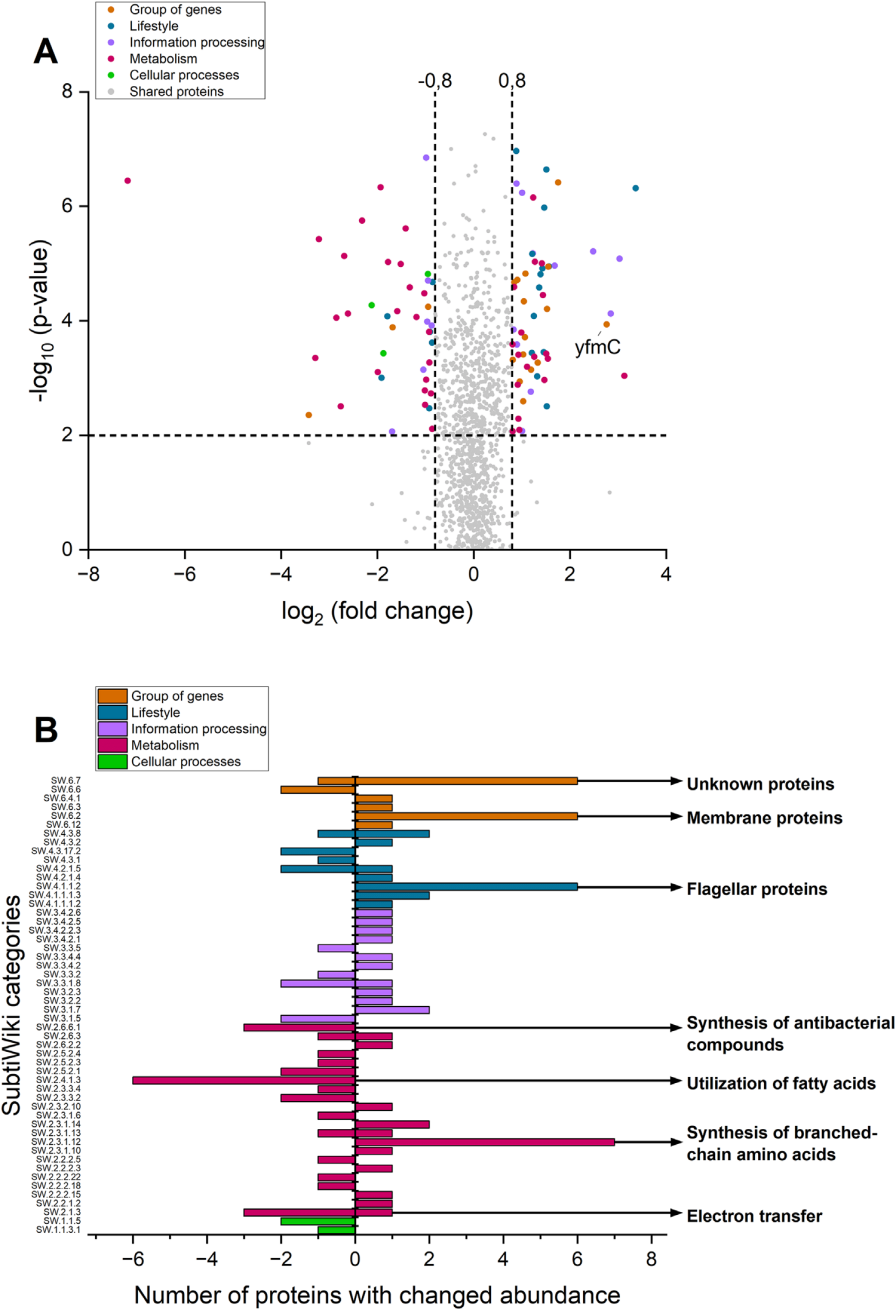
Proteome analysis of γ‐PGA‐producing cells cultivated in the presence of 1 or 15 g L^−1^ of citrate. The proteome of *B. subtilis* PG10 harvested after 48 h of cultivation in a medium with 1 (low) or 15 (high) g L^−1^ citrate is shown (biological triplicates). The experiment was performed in 250 mL shake flasks with 30 mL filling volume, at 300 rpm (25 mm shaking diameter) and 37°C. The data is visualized as volcano plot (A), hits after a Student *t*‐test (*p* value < 0.01), and log_2_ fold changes >0.8 or <–0.8 are highlighted according to their respective SubtiWiki category. A log_2_ fold change >0.8 resembles higher abundance during high citrate availability, whereas a log_2_ fold change of <–0.8 shows higher abundance in the low citrate condition. The number of proteins with altered abundance per category is shown as a bar chart (B), where categories containing ≥3 differentially abundant proteins are labeled specifically.

To further characterize the metabolic state of γ‐PGA producers while facing changes in citrate availability, the proteome of these cells was analyzed, revealing significant differences in protein accumulation and depletion. The respective proteins were grouped according to their allocated protein category displayed in the SubtiWiki database and thereby classified as *Group of genes*, *Lifestyle*, *Information processing*, *Metabolism* and *Cellular processes*. A full list of proteins with significant changes in protein abundance can be found in the Supplementary Materials. In general, a great variety of proteins, involved in different cellular functions were detected based on the presence or absence of citrate. Proteins predominantly accumulated after cultivation with low citrate availability include all proteins of fatty acid (FA) degradation, including enzymes for electron transfer, FA activation, and co‐factor regeneration (LcfAB, EtfAB, Fad(E/H/B/N/A)). Moreover, the accumulation of proteins involved in the synthesis of the glutamate‐containing antibacterial compound kanosamine (NtdABC) was identified (Figure 4A). On the other hand, cultivation in the presence of high citrate concentrations led to, together with a variety of other proteins, the accumulation of six flagellar proteins (Hag, Fli(M/G/H/F), FlgG). Proteins involved in the biosynthesis of branched‐chain amino acids (Ilv(B/C/H), Leu(A/B/C)) were also more abundant, together with unknown‐ and membrane proteins. One of these membrane proteins, YfmC, is annotated as the binding domain of Fe(III)/citrate clusters and belongs to the FecCDEF iron/citrate transporter system. However, only YfmC of the four proteins building the whole transportation apparatus was detected. FecD and FecE both contain membrane‐spanning domains, suggesting that the proteins were not sufficiently extracted with the applied protocol. Since the components are organized in an operon, it is however likely, that the complete transporter system was produced at elevated levels. As the accumulation of this protein belonging to a citrate transporter was observed during citrate surplus, this protein complex may be of importance upon further analysis of citrate‐mediated regulation. Moreover, the presence of this transporter might facilitate minor Fe/citrate influx, thereby influencing intracellular iron homeostasis. Consequently, it was investigated in how far the transporter may be involved in the effect of citrate availability on γ‐PGA synthesis.

### 3.4 γ‐PGA Synthesis of *B. subtilis* PG10 Mutants With Deletions of Genes Involved in Citrate Sensing and Transportation

**FIGURE 5:**
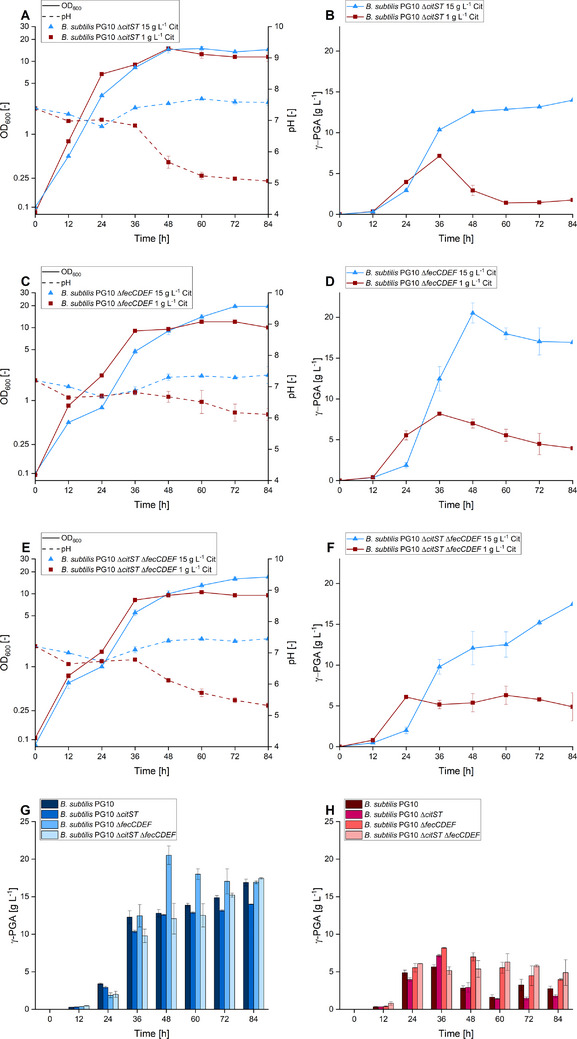
Quantitative physiology and γ‐PGA titer of *B. subtilis* deletion mutants in the presence of different citrate concentrations. Growth, pH development, and progression of γ‐PGA titer of γ‐PGA producing *B. subtilis* deletion strains deficient in citrate‐sensing or transport in the presence of 20 g L^−1^ glutamate and high (15 g L^−1^) or low (1 g L^−1^) levels of exogenous citrate (Cit) is shown. In this experiment, the strains *B. subtilis* Δ*citST* (A, B), *B. subtilis* Δ*fecCDEF* (C, D) and the double deletion mutant *B. subtilis* Δ*citST* Δ*fecCDEF* (E, F) were tested. The biopolymer produced by the deletion mutants is further compared to the production dynamics of the parental strain in the presence of 15 g L^−1^ citrate (G) or 1 g L^−1^ citrate (H). The experiment was performed in 500 mL shake flasks with 50 mL filling volume, at 300 rpm (25 mm shaking diameter) and 37°C. The experiments were performed in duplicates, error bars represent the deviation from the average.

A recent study provided evidence that the response regulator CitT not only regulates citrate uptake but also the expression of off‐targets such as genes encoding enzymes involved in pectin degradation [42]. To investigate if the two‐component system CitST is involved in citrate sensing and thereby transmits a signal to the cell which is not limited to transport and thus influences γ‐PGA synthesis, the *citST* operon was deleted, yielding strain *B. subtilis* Δ*citST*. Based on the finding of the proteome analysis that elevated citrate availability led to the accumulation of YfmC, a component of the Fe(III)‐citrate transporter FecCDEF, the whole operon was deleted in *B. subtilis* 168, yielding strain *B. subtilis* Δ*fecCDEF*. Last, a double deletion mutant of *citST* and *fecCDEF* was constructed to investigate if CitST can contribute to the regulation of other citrate transporters apart from CitM, ultimately yielding strain *B. subtilis* Δ*citST* Δ*fecCDEF*. Regarding growth behavior, all tested strains showed a similar trend of a faster increase in OD_600_ in the first 36 h when facing low exogenous citrate (Figure 5). From 48 h onward, the OD_600_ detected in the low citrate condition was surpassed by cells grown in the presence of high citrate availability, consistent in all tested strains. Whereas the pH is mostly constant throughout cultivation in the presence of high citrate concentrations, a gradual pH decrease was observed in the case of low citrate availability after 36 h, resembling the point of growth arrest. This trend was visible in all tested strains but was more prominent upon deletion of *citST*. In the case of γ‐PGA synthesis, high citrate availability resulted in a polymer titer of 12.6 g L^−1^ after 48 h in *B. subtilis* Δ*citST*, followed by no further significant increase in the secreted product throughout the remainder of the experiment. *B. subtilis* Δ*fecCDEF* produced a maximum polymer titer of 20.5 g L^−1^ also after 48 h of cultivation. Afterward, the titer gradually decreased to 16.9 g L^−1^ at the end of the experimental run. In both latter strains, most polymer synthesis occurred within the first 48 h of the experiment. However, upon the double deletion of *citST* and *fecCDEF*, this trend was altered toward a continuous product synthesis throughout the whole cultivation, reaching a final γ‐PGA titer of 17.4 g L^−1^ after 84 h. When only little citrate was supplied to the cultivation, the titer continuously increased within the first 36 h of cultivation, reaching a titer of 7.1 g L^−1^ for *B. subtilis* Δ*citST* and 8.2 g L^−1^ for *B. subtilis* Δ*fecCDEF*. The double deletion strain reached a maximum titer of 6.1 g L^−1^ after 24 h. Afterward, the γ‐PGA titer synthesized by the single deletion strains decreased gradually until the end of cultivation, whereas it remained constant in the case of the double deletion strain. Citrate consumption was not altered by the deletions and followed the same trend as depicted in Figure 1 (Data not shown). Based on the available data we expected a decrease in product titer in the presence of high citrate concentrations upon deletion of genes that are involved in citrate sensing and transport. Contradictory, no significant change in production dynamic was observed among the deletion and the parental strain (see Figure 5G,H). Notably, the deletion of *fecCDEF* and therefore the absence of the Fe(III)‐citrate transporter increased product synthesis in the first 48 h of the experiment, indicating the presence of further networks involved in citrate sensing and transport.

## 4 Discussion

The dependency of γ‐PGA synthesis on the supply of exogenous glutamate is an important cost‐ and performance‐defining factor for industrialization [43]. One option to improve glutamate‐independent γ‐PGA production is to increase glutamate de novo synthesis in the production host. In this context, Zhang et al. have reported a co‐feed of glucose and either citric acid or oxalic acid to improve glutamic acid synthesis and subsequently γ‐PGA production in *B. subtilis* [44]. The authors identified that citrate stimulated both the activity of the glutamate‐ and isocitrate dehydrogenase, thereby incorporating internalized citrate into the TCA cycle, serving as a precursor for α‐ketoglutarate and subsequent glutamate synthesis. In another study, a glucose‐citrate co‐feed was shown to improve D‐ribose production in *B. subtilis* by altering the specific activity of various enzymes involved in central carbon metabolism. Consequently, the co‐feed resulted in increased levels of NADPH and decreased glycolytic flux [45]. Remarkably, the authors contextualized the findings with an increase in intracellular metal‐ion content. Importantly, these mechanisms of an interplay between citrate supplementation and altered protein activity rely on the premise that citrate is internalized. The above‐mentioned studies demonstrated citrate uptake into *B. subtilis* in the presence of glucose. However, citrate import is impeded by CCR in *B. subtilis* 168, which is why most of the supplemented citrate was not taken up by the cell during the cultivations presented in this work. The initial decrease in citrate concentration between 24 and 36 h may be subjected to transport by other poorly described citrate transporters such as CitH or similar di/tricarboxylate transporters. Since an equal inflow of citrate into the cell, irrespective of the initial exogenous citrate level was observed, it is unlikely that the variations in growth behavior and biopolymer production are the result of direct alterations in gene expression induced by altered intracellular citrate/metal‐ion homeostasis but rather rely on a different regulatory framework. In *E. coli*, citrate stimulates not only the dedicated CitAB two‐component system but also DcuSR, which is involved in co‐sensing C4‐dicarboxylates and citrate [46]. The closely related *B. subtilis* two‐component system DctSR is also known to be stimulated by C4‐dicarboxylates, but involvement in citrate co‐sensing has so far not been demonstrated. Future research will be necessary to show how far this sensor chassis can be induced by citrate and if it has an indirect effect on γ‐PGA production.

Glutamate uptake was comparable in strains facing either high or low exogenous citrate concentration and in the case of the latter, the available glutamate was even consumed faster. This raises the question of why glutamate is efficiently internalized irrespective of citrate availability, but the observed product titer differs significantly. This could indicate that mere citrate availability might control a yet unknown regulatory network, which is involved in governing intracellular glutamate homeostasis. Since glutamate is regarded as a branching point between carbon‐ and nitrogen‐metabolism, strict control of intracellular glutamate levels is crucial for microbial fitness [47]. In this context, low levels of exogenous citrate might accelerate cellular glutamate‐consuming reactions. Thus, less glutamate is available for γ‐PGA synthesis. This hypothesis is supported by the findings of the performed proteome analysis. In the presence of low exogenous citrate availability, proteins involved in kanosamine synthesis, a glutamate‐containing antibacterial compound, were more abundant. The activation of fatty acids by the addition of acetyl CoA catalyzed by LcfAB is also involved as an initial step in the production of surfactin, a glutamate‐containing surface‐active molecule [48].

One possible inductor of these alternative glutamate‐consuming pathways may be posed by the change in medium viscosity depending on the used amount of citrate. In *B. subtilis* 168, medium viscosity can be sensed through flagellar drag, resulting in DegSU‐mediated cell differentiation [49]. A sudden increase in medium viscosity may induce colony differentiation into, for example, the K state or state of bacterial competence, which involves major changes in bacterial metabolism [50]. This hypothesis is further supported by the results of this work, as the viscosity spike coincided with production arrest. Moreover, the proteome analysis presented here which was based on samples obtained after the viscosity spike (48 h) revealed a depletion in flagellar bodies. During biofilm formation, *B. subtilis* secrets exopolysaccharides, subsequently increasing the viscosity and triggering the transition into becoming sessile [51]. The viscosity increase observed upon citrate depletion might confer a similar signal as upon biofilm formation, resulting in further changes in metabolism. To explain why the medium viscosity changed depending on citrate availability, we assume that depletion of a suitable chelator during cultivation with low citrate resulted in the exposition of free divalent cations such as Mg^2+^, Ca^2+^, or Mn^2+^. Consequently, electrostatic interactions between the dissociated carboxylic acid groups of the biopolymer or the negatively charged cell surface of the microbe and the metal ions might promote gelation of the cultivation medium, increasing the overall viscosity. In the case of calcium, the progression of gelation in combination with carboxylic acids has already been studied in detail, and a high electrostatic affinity towards γ‐PGA has also been demonstrated [52, 53]. In case of high extracellular citrate levels, excess cations are likely chelated by citrate, thereby preventing potential interactions with the biopolymer and reducing the overall viscosity profile during production. Indeed, a lower online viscosity was observed in the presence of high citrate levels in comparison to the viscosity attained by *B. licheniformis* at a comparable titer and even lower molecular weight [40]. The stagnating OTR signal indicates oxygen limitations during the synthesis of the biopolymer, which has also been depicted in previous studies [26, 29, 40]. Interestingly, a previous study has postulated that an oxygen limitation leads to the accumulation of 2‐oxoglutarate due to an NADH surplus, which potentiated γ‐PGA synthesis in *B. licheniformis* [24]. This effect was observed for viscosities <20 mPa*s which are significantly lower as observed in this work. Thus, we hypothesize that a beneficial effect of oxygen limitation on γ‐PGA synthesis only occurs until a certain threshold, as very high viscosities of >100 mPa*s were associated with production arrest.

To improve the economic feasibility of a γ‐PGA production process, different studies have highlighted the role of (industrial) waste streams as nutrient sources. Depending on the medium and setup used for cultivation, titers between 5.4 and 112.8 g L^−1^ were reached by providing additional exogenous glutamate [3, 4]. In this study, a maximum γ‐PGA titer of 20.5 g L^−1^ was reached and was directly correlated to exogenous glutamate availability. High citrate concentrations were necessary to control medium viscosity and ensure conversion of the glutamate feed to γ‐PGA. With this, the presented study offers valuable insight into how to improve glutamate conversion into γ‐PGA, thus supporting future approaches of cost‐reduction upon waste stream utilization. Future work will also focus on elucidating novel citrate‐sensing and transportation mechanisms with special regard to potential regulatory frameworks, induced by citrate. Here, the interplay between citrate and glutamate will be investigated, as a profound understanding of regulatory networks spanned around glutamate metabolism is of particular importance to guide future optimization approaches of γ‐PGA synthesis. To our knowledge, this is the first study demonstrating that the medium viscosity during γ‐PGA production is not necessarily dependent on polymer titer and molecular weight, but other medium components such as citrate and its interaction with divalent cations have to be considered as well. Consequently, quantitative evaluation and comparison of viscosity data to mimic biopolymer production is only valid for constant media composition.

## Author Contributions

F.V. conceptualized the study, performed the experiments, evaluated the data, prepared the original draft, and revised the manuscript. K.H. performed the online/offline viscosity analysis, OTR measurements, molecular weight analysis, and revised the manuscript. S.M. performed the proteome analysis and revised the manuscript. B.H.M. conceptualized the study and revised the manuscript. L.M.B. conceptualized the study, provided supervision, acquired funding, and revised the manuscript. J.B. revised the manuscript.

## Conflicts of Interest

The authors declare no conflicts of interest.

## Supporting information



Supporting Information

## Data Availability

The physiological data that support the findings of this study are available from the corresponding author upon reasonable request. Proteome data are openly available in the ProteomeXchange Consortium via the PRIDE partner repository with the dataset identifier PXD058048.
