# Supplementary material for: Citrate Supplementation Modulates Medium Viscosity and Poly‐γ‐Glutamic Acid Synthesis by Engineered B. subtilis 168
Source: Eng Life Sci. 2025 Mar 4;25(3):e70009. doi: 10.1002/elsc.70009 (PMC11880625; doi:10.1002/elsc.70009)
Supplement: Supplementary file 1 — Supporting Information [file ELSC-25-e70009-s001.docx]

| **Protein name** | **Log_2_ fold change > 0.8** | **SubtiWiki category** | **Protein name** | **Log_2_ fold change < -0.8** | **SubtiWiki category** |
| --- | --- | --- | --- | --- | --- |
| Hag | 3.364 | SW.4.1.1.2 | PhoB | -7.187 | SW.2.6.3 |
| AroH | 3.127 | SW.2.3.1.13 | YncM | -3.424 | SW.6.7 |
| Nin | 3.030 | SW.3.1.7 | LcfA | -3.289 | SW.2.4.1.3 |
| ComEB | 2.845 | SW.3.1.7 | FadE | -3.214 | SW.2.4.1.3 |
| YfmC | 2.759 | SW.6.2 | FadA | -2.851 | SW.2.4.1.3 |
| RapH | 2.480 | SW.3.3.4.4 | NtdB | -2.762 | SW.2.6.6.1 |
| YfhC | 1.753 | SW.6.7 | FadN | -2.689 | SW.2.4.1.3 |
| DbpA | 1.682 | SW.3.2.3 | FadH | -2.612 | SW.2.4.1.3 |
| FlgG | 1.561 | SW.4.1.1.2 | NtdA | -2.319 | SW.2.6.6.1 |
| Maf | 1.548 | SW.6.7 | YoeB | -2.118 | SW.1.1.3.1 |
| LeuA | 1.540 | SW.2.3.1.12 | GuaC | -1.991 | SW.2.5.2.3 |
| YchF | 1.524 | SW.6.3 | EtfA | -1.934 | SW.2.1.3 |
| YdhF | 1.518 | SW.4.2.1.4 | YobL | -1.914 | SW.4.3.17.2 |
| YfmS | 1.510 | SW.4.1.1.1.3 | WapA | -1.877 | SW.1.1.5 |
| PckA | 1.506 | SW.2.2.1.2 | YwqJ | -1.792 | SW.4.2.1.5 |
| IlvB | 1.467 | SW.2.3.1.12 | EtfB | -1.778 | SW.2.1.3 |
| FliG | 1.465 | SW.4.1.1.2 | UvrB | -1.696 | SW.3.1.5 |
| YozE | 1.453 | SW.4.2.1.5 | YhcB | -1.684 | SW.6.6 |
| LeuD | 1.437 | SW.2.3.1.12 | XynA | -1.588 | SW.2.2.2.5 |
| FliM | 1.426 | SW.4.1.1.2 | FadB | -1.516 | SW.2.4.1.3 |
| YumC | 1.415 | SW.2.1.3 | UreB | -1.412 | SW.2.3.3.2 |
| HemAT | 1.387 | SW.4.1.1.1.3 | UreC | -1.327 | SW.2.3.3.2 |
| fFiH | 1.361 | SW.4.1.1.2 | YkfD | -1.189 | SW.2.3.3.4 |
| YjcQ | 1.329 | SW.6.7 | UvrA | -1.045 | SW.3.1.5 |
| YdjO | 1.318 | SW.4.3.2 | GlvA | -1.026 | SW.2.2.2.22 |
| LeuC | 1.271 | SW.2.3.1.12 | TrpE | -1.015 | SW.2.3.1.13 |
| LeuB | 1.257 | SW.2.3.1.12 | BglH | -1.011 | SW.2.2.2.18 |
| MsrB | 1.250 | SW.4.3.8 | YukC | -0.987 | SW.3.3.5 |
| GabT | 1.234 | SW.2.3.2.10 | CypE | -0.987 | SW.2.1.3 |
| CheV | 1.227 | SW.3.4.2.2.3 | YrrO | -0.966 | SW.3.3.1.8 |
| MsrA | 1.216 | SW.4.3.8 | YkfB | -0.950 | SW.1.1.5 |
| FliF | 1.207 | SW.4.1.1.2 | YrrN | -0.948 | SW.3.3.1.8 |
| YfkN | 1.190 | SW.6.12 | AmhX | -0.947 | SW.6.6 |
| YdeE | 1.185 | SW.3.4.2.6 | PurA | -0.925 | SW.2.5.2.1 |
| IlvH | 1.108 | SW.2.3.1.12 | KatA | -0.923 | SW.4.3.8 |
| YxkC | 1.073 | SW.6.7 | YyrG | -0.921 | SW.2.5.2.4 |
| McpA | 1.064 | SW.6.2 | YeeF | -0.906 | SW.4.3.17.2 |
| YmcC | 1.043 | SW.6.7 | PurK | -0.887 | SW.2.5.2.1 |
| YvrO | 1.029 | SW.6.2 | GroL | -0.873 | SW.3.3.2 |
| YneF | 1.027 | SW.6.2 | katE | -0.864 | SW.4.3.1 |
| CheY | 1.007 | SW.3.4.2.1 | yszB | -0.863 | SW.2.3.1.6 |
| CspC | 1.005 | SW.3.2.2 | ywqK | -0.854 | SW.4.2.1.5 |
| MtnK | 0.990 | SW.2.3.1.10 |  |  |  |
| YwpJ | 0.955 | SW.6.4.1 |  |  |  |
| HisK | 0.948 | SW.2.3.1.14 |  |  |  |
| RibE | 0.929 | SW.2.6.2.2 |  |  |  |
| PhoD | 0.928 | SW.2.6.3 |  |  |  |
| IlvC | 0.914 | SW.2.3.1.12 |  |  |  |
| McpC | 0.905 | SW.6.2 |  |  |  |
| OpcR | 0.898 | SW.3.4.2.5 |  |  |  |
| CheA | 0.888 | SW.3.3.4.2 |  |  |  |
| CheW | 0.883 | SW.4.1.1.1.2 |  |  |  |
| McpB | 0.843 | SW.6.2 |  |  |  |
| HisH | 0.839 | SW.2.3.1.14 |  |  |  |
| TrmFO | 0.829 | SW.3.3.1.8 |  |  |  |
| YurQ | 0.811 | SW.6.7 |  |  |  |
| GlpQ | 0.810 | SW.2.2.2.3 |  |  |  |
| ExuR | 0.806 | SW.2.2.2.15 |  |  |  |
